# Supplementary material for: Robust Generation of Topological Biphoton Mode via Adiabatic Passage
Source: Nanophotonics. 2026 Jul 29;15(15):e70223. doi: 10.1002/nap2.70223 (PMC13642563; doi:10.1002/nap2.70223)
Supplement: Supplementary file 1 [file NAP2-15-e70223-s001.zip › Supplement_R1_clean.pdf]

Supporting Information for "Robust Generation of Topological Biphoton Mode via Adiabatic Passage"

Jaesung Lim, Jihwan Kim, Dong-Gil Im, Kyungdeuk Park, Dongkyu Kim, Yonggi Jo, and Yong Sup Ihn*
Agency for Defense Development, Daejeon 34186, Korea
(Dated: June 23, 2026)

S1. PRACTICAL PERFORMANCE UNDER FABRICATION IMPERFECTIONS

While the main text characterizes the topological biphoton generation by computing the fidelity with respect to the disorder-dependent topological biphoton mode of each sample, experimental assessment and practical applications often require a comparison against the ideal, disorder-free mode. Furthermore, in realistic devices, fabrication imperfections arise not only from gap variations but also from waveguide width fluctuations. The width imperfections modify the on-site (diagonal) terms of the Hamiltonian, breaking the chiral symmetry and removing the corresponding topological protection. In this section, we analyze the fidelity relative to the ideal topological biphoton state under both types of disorder to evaluate the practical performance.

Fig. S1(a) shows the biphoton fidelity in the presence of gap disorder for both adiabatic and conventional topological waveguide arrays with pump injection into the central waveguide. The black data points represent the mean fidelity between the topological biphoton mode of each disordered sample and the ideal mode. As the coupling constants are modified by gap disorder, the eigenmode profile deviates from the ideal form, leading to a gradual decrease in fidelity, with an average value of 0.905. In the adiabatic structure, the topological biphoton mode is generated for each sample, with the fidelity reflecting this disorder-induced mode distortion and decreasing to 0.896. However, the conventional array, which suffers from the admixture of non-topological components, shows a decline to 0.768, demonstrating a clear performance difference.

We next examine the impact of simultaneous diagonal and off-diagonal disorder. The diagonal disorder is introduced through variations in the propagation constants arising from waveguide width perturbations. Specifically, Gaussian noise is added to the waveguide width with a standard deviation δ', varied from 0 to 2 nm in steps of 0.4 nm. The resulting changes in the propagation constants are incorporated into the diagonal terms of the Hamiltonian. The off-diagonal disorder is implemented in the same manner as above, with the disorder strength of $\delta = 40$ nm. Fig. S1(b) compares the fidelities of the adiabatic structure and the topological waveguide array. In the absence of width disorder, the fidelity difference of 0.128 is observed. As δ' increases, the broken chiral symmetry reduces the mean fidelity in both cases. While the performance gap between the two structures narrows to 0.077, the advantage of the adiabatic scheme remains clear. These results confirm that the adiabatic passage is a superior strategy for high fidelity quantum state preparation in realistic devices where multiple sources of disorder coexist.

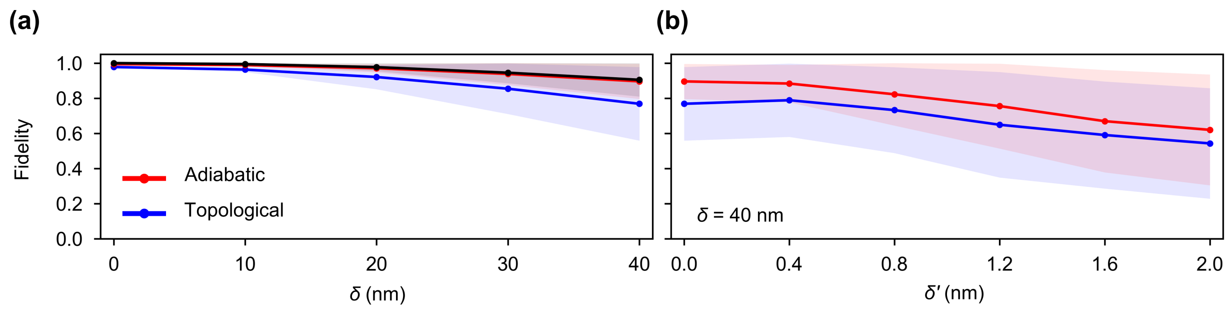

FIG. S1. Practical performance evaluation of topological biphoton generation. (a) Output biphoton mode fidelity with respect to the ideal mode across different gap disorder strengths for the adiabatic (red) and topological (blue) structures. The black points indicate the fidelity between the disordered topological biphoton mode of each sample and the ideal mode, representing the gap disorder-induced mode distortion. (b) Output biphoton mode fidelity relative to the ideal mode under increasing width disorder strength for both structures. The gap disorder with a strength of $\delta = 40$ nm is simultaneously introduced.

* yong0862@add.re.kr
